# OHNOLOGS v2: a comprehensive resource for the genes retained from whole genome duplication in vertebrates

**DOI:** 10.1093/nar/gkz909

**Published:** 2019-10-15

**Authors:** Param Priya Singh, Hervé Isambert

**Affiliations:** Institut Curie, Research Center, CNRS UMR168, PSL Research University, 26 rue d’Ulm, 75005, Paris, France

## Abstract

All vertebrates including human have evolved from an ancestor that underwent two rounds of whole genome duplication (2R-WGD). In addition, teleost fish underwent an additional third round of genome duplication (3R-WGD). The genes retained from these genome duplications, so-called ohnologs, have been instrumental in the evolution of vertebrate complexity, development and susceptibility to genetic diseases. However, the identification of vertebrate ohnologs has been challenging, due to lineage specific genome rearrangements since 2R- and 3R-WGD. We previously identified vertebrate ohnologs using a novel synteny comparison across multiple genomes. Here, we refine and apply this approach on 27 vertebrate genomes to identify ohnologs from both 2R- and 3R-WGD, while taking into account the phylogenetically biased sampling of available species. We assemble vertebrate ohnolog pairs and families in an expanded OHNOLOGS v2 database. We find that teleost fish have retained more 2R-WGD ohnologs than mammals and sauropsids, and that these 2R-ohnologs have retained significantly more ohnologs from the subsequent 3R-WGD than genes without 2R-ohnologs. Interestingly, species with fewer extant genes, such as sauropsids, have retained similar or higher proportions of ohnologs. OHNOLOGS v2 should allow deeper evolutionary genomic analysis of the impact of WGD on vertebrates and can be freely accessed at http://ohnologs.curie.fr.

## INTRODUCTION

Gene duplication provides raw material for the evolution of new gene functions (1). Duplication of single genes or genomic segments is a continuous evolutionary process that creates diversity in terms of copy number variations across individuals, and paralogs across species. In addition, dramatic evolutionary accidents corresponding to whole genome duplication (WGD) have also occurred in the evolutionary past of most eukaryotic lineages including plans, fungi and animals (2–4). For example, all extant vertebrates have experienced two rounds of WGDs (2R-WGD) in their evolutionary past (5–8). In addition, a third round of genome duplication has also occurred in the teleost fish lineage (3R-WGD) (9–11). 2R-WGDs likely played important roles in the evolution and diversification of vertebrate specific innovations such as neural crest cells, placodes and a complex brain (12,13). Many key genes implicated in the development of these structures can be traced back to 2R-WGD. Similarly, 3R-WGD likely played an important role in the expansion of the diversity of teleost fish lineage making it the most species rich vertebrate group (14–17). Hence, the genes retained from these three WGD events have been instrumental in the evolution of vertebrates (18).

The genes originated from these ancient polyploidy (paleo-polyploidy) events are now called ohnologs after Susumu Ohno who first hypothesized the two rounds of WGD events in vertebrate ancestors (1,5,19). Ohnologs are known to have distinct evolutionary, genomic and functional properties that distinguish them from small-scale duplicates and singletons (20–23). They also show greater association with diseases and cancer than non-ohnolog genes (24–29), and have been suggested to be dosage balanced (24), which was subsequently argued to be indirectly mediated by their high susceptibility to dominant mutations (25,28), as supported by quantitative population genetics models (27) and by a global inference approach assessing direct *versus* indirect causal relationships across multiple genomic properties (30).

Given the specific impact WGDs have had on the evolution of vertebrates, a comprehensive database of vertebrate ohnologs is highly desirable. While there are some useful resources available for comparison of synteny across species (31–34) there is no database that reliably identifies ohnologs from both vertebrate 2R-WGDs and fish 3R-WGD. To start filling this gap, we developed in 2015, OHNOLOGS, a repository of ohnologs retained from the 2R-WGD in six amniote vertebrates (human, mouse rat, pig, dog and chicken) (34). OHNOLOGS is based on a novel comparative macro-synteny approach that reliably identifies ohnologs, despite lineage specific genome rearrangement, gene loss and small scale duplication events, by combining macro-synteny information (gene content regardless of exact order) across multiple outgroups and vertebrate genomes (34).

Here, we expand this multiple genome synteny comparison approach to 27 vertebrate species including four teleost fish species. We further improve the statistical confidence assessment of each ohnolog pair with a weighted quantitative confidence score (q-score) taking into account the phylogenetically biased sampling of available vertebrate species. In addition, we uncover ohnologs, including in non-protein coding RNA gene classes, from both 2R-WGD in early vertebrates (2R-ohnologs) and 3R-WGD in teleost fish (3R-ohnologs). The expanded OHNOLOGS database is the most comprehensive repository of ohnologs in vertebrates. Using the new OHNOLOGS database we show that on average 25% of extant genes are 2R-ohnologs in vertebrates and that 18% of extant genes are 3R-ohnologs in teleost fish. Sauropsids show the highest lineage-specific loss of 2R-ohnologs, and teleost fish show the highest lineage specific retention of 2R-ohnologs. We also found that 2R-ohnologs are significantly more likely to retain 3R-ohnologs in teleost fish, in agreement with earlier reports (35). OHNOLOGS v2 should facilitate deeper evolutionary analysis of the unique properties of ohnologs, and their lineage-specific retention and loss in different vertebrates.

## RESULTS

### Data collection and processing

**Figure 1.**
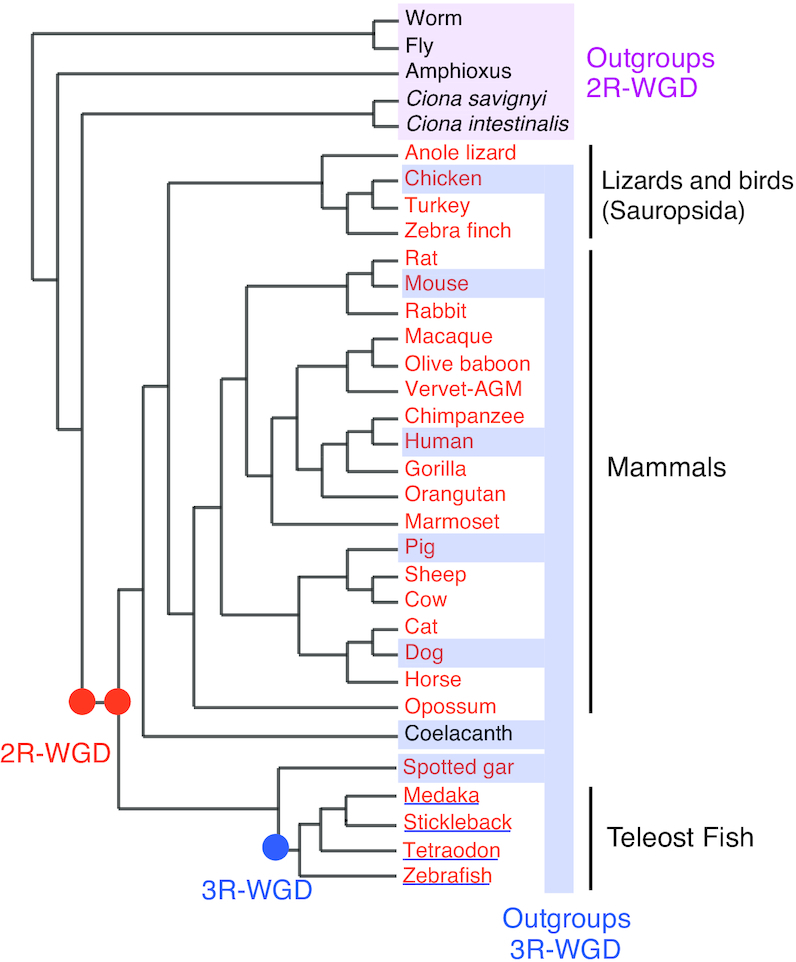
A schematic phylogeny (not scaled) of the organisms in the OHNOLOGS v2 database. Vertebrates analysed for 2R-WGD are in orange, and teleost fish species analysed for 3R-WGD are underlined. Outgroup species used to identify 2R- and 3R-ohnologs have been highlighted.

OHNOLOGS v2 includes 2R-ohnolog pairs and families in 27 vertebrates that have a chromosome level assembly with a majority of their genes anchored on chromosomes in Ensembl version 84 (36). This includes 18 mammals, 4 sauropsids (lizards and birds), 4 teleost fish and spotted gar. In addition, we also included 3R-ohnolog pairs and families in four teleost fish genomes. We used five non-vertebrate outgroups to identify 2R-ohnologs and seven vertebrate outgroups to identify fish specific 3R-ohnologs (Figure 1 and Supplementary Table S1).

We collected genes (protein coding, micro-RNA, miscellaneous RNA, rRNA, snRNA and snoRNA) for all these organisms from Ensembl v84 using biomaRt (37,38). These six classes of genes were chosen because they have information on orthologs and paralogs across many vertebrates. Orthologs, paralogs and relative duplication node for all the genes were obtained from Ensembl comparative genomics resource (39). These homology relationships and their relative duplication time have been computed by reconciling gene based phylogenetic trees with the species phylogeny for each Ensembl gene family (40). To identify duplication time of paralogs consistently, we took the consensus timing across 7 Ensembl versions (v80–v86). Genes with a lot of small-scale duplications (>30), which inflate the synteny calculations, were excluded from analysis. Genome data for Amphioxus was obtained from JGI and amphioxus orthologs with other organisms were identified using BLASTp (8).

We adapted the macro-synteny comparison approach, previously developed in (34), to identify ohnologs retained from both 2R-WGD (2R-ohnologs) and 3R-WGD (3R-ohnologs). Briefly, for each pair of outgroup and paleo-polyploid organisms, we first identified blocks of conserved macro-synteny using windows ranging from 100 to 500 genes (outgroup comparison). These macro-synteny blocks have a pattern of doubly conserved synteny, where a window in the outgroup genome shares orthology with at least two other windows in the paleo-polyploid genome. The paralogs residing on these windows and duplicated at the time of 2R- or 3R-WGD are candidates for being 2R- or 3R-ohnologs, respectively. Similarly, we also identified syntenic windows by comparing each paleo-polyploid genome to itself (self comparison).

To refine these ohnologs further and eliminate spurious synteny patterns, we computed a quantitative score (called q-score) to assess the probability that any ohnolog pair could be identified by chance, following the approach developed in (34). In brief, all q-scores from different windows and outgroups were combined to give a global q-score for each ohnolog pair from outgroup comparison. Using multiple outgroups allowed us to identify ohnologs that may have moved to non-syntenic locations in some of the outgroup genomes. Similarly we obtained a q-score for self comparison to assess the chance of spurious association. In addition, while we used a simple geometric average of q-scores in (34), which cannot capture the gain of statistical power expected from the integration of multiple vertebrate genomes, here we developed a refined weighting scheme of species, which also takes into account the strong phylogenetically biased sampling of included species by using different weights for each vertebrate genome depending on its shared homology with other included genomes (see Supplementary Methods for details, Supplementary Tables S2 and 3).

Using both self and outgroup weighted q-scores, we generated three sets of ohnologs (corresponding to strict, intermediate and relaxed criteria) and combined them into ohnolog families. At last, we compiled both the 2R- and 3R-ohnolog pairs and ohnolog families for each organism in the interactive OHNOLOGS v2 database using Apache, CGI, Perl, Bootstrap and jQuery.

### Navigating the OHNOLOGS database

**Figure 2.**
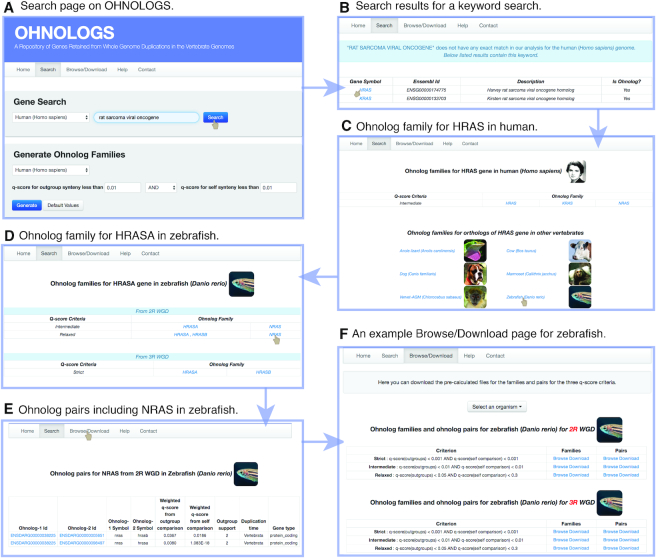
Navigating the OHNOLOGS database. (**A**) Screenshot of the search page. (**B**) Result page for a keyword search of ‘rat sarcoma viral oncogene’ shows the matching genes in human. (**C**) Ohnolog family page for HRAS gene in the human genome. (**D**) From the family page, users can navigate to ortholog families in other vertebrates, e.g. zebrafish HRASA. (**E**) Ohnolog pair page for zebrafish for NRAS gene. (**F**) Browse/Download page for zebrafish showing both 2R and 3R-ohnolog pairs and families for all the three criteria.

The home page lists all the organisms that are included in OHNOLOGS for 2R and 3R-WGD along with an introduction on ohnologs and WGDs. The search page allows a user to search for a gene symbol, Ensembl Id GO term or any keyword (Figure 2A). The search page also allows one to generate ohnolog families for any user-defined q-score criteria for a given organism. Upon a keyword or GO term query, all matching genes will be displayed along with their ohnolog status (Figure 2B). If a queried gene is an ohnolog, its ohnolog family will be displayed on the result page (for both 2R and 3R WGD for teleost fish) (Figure 2C and D). We show families for our strict q-score filter, and display the intermediate and relaxed families only if additional ohnologs are identified upon relaxing the q-score filter. The result page also includes links to pair page that has all ohnolog pairs that went into constructing that family (Figure 2E). The family result pages also links to the orthologous genes and ohnolog families in other vertebrates, to study the conservation of ohnolog families in other vertebrates.

The ohnolog pairs and families for our three pre-defined q-score filters can be explored and downloaded from the Browse/Download pages (Figure 2F). We link the genes on the browse pages to external databases including Ensembl, NCBI gene, GeneCards (for human), MGI (for mouse) and ZFIN (for zebrafish). The details of our approach, family descriptions and more details on q-score have also been included on the help page.

### Summary of the contents of the OHNOLOGS database

**Figure 3.**
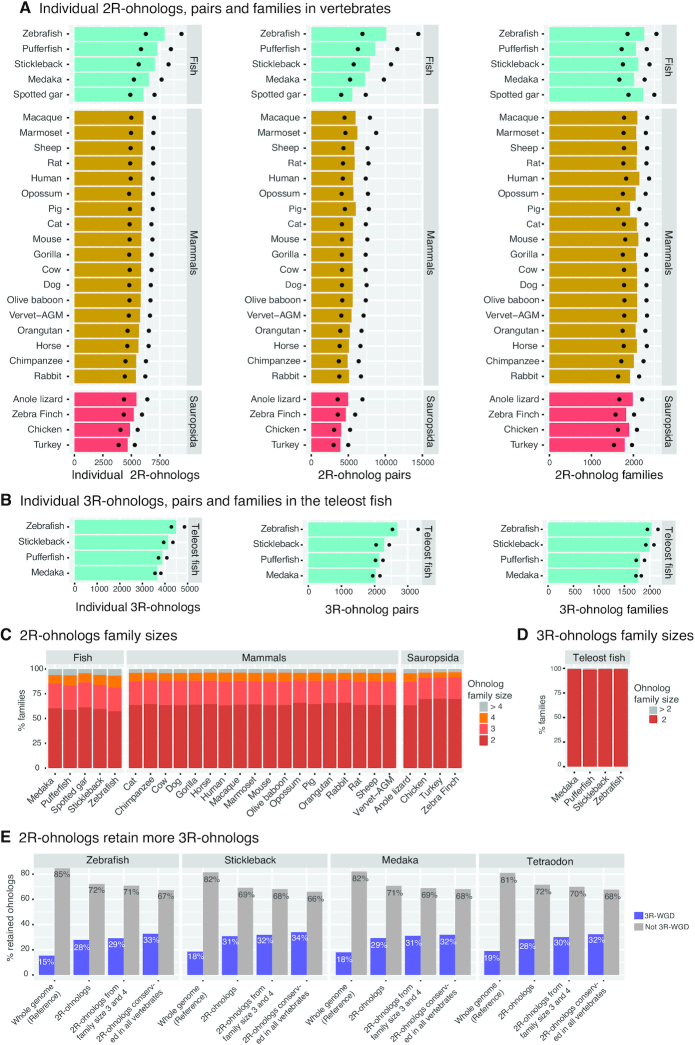
Description of the ohnolog genes, pairs and families in the database. (**A**) Number of retained individual 2R-ohnolog genes, pairs and families in all the 27 vertebrates. Bars represent the numbers from the intermediate criterion. Ohnologs from strict and relaxed criteria are indicated by dots. (**B**) Number of retained individual 3R-ohnolog genes, pairs and families in the four teleost fish species. Bars represent the numbers from the intermediate criterion. Ohnologs from strict and relaxed criteria are indicated by dots. (**C**) Size of the 2R-ohnolog families from the intermediate criterion in vertebrates. Note that a vast majority of the families are of size 2, 3 or 4. (**D**) Sizes of the 3R-ohnolog families from the intermediate criterion in the teleost fish hardly exceed size two. (**E**) The 2R-ohnologs are significantly more likely to retain 3R-ohnologs, compared to genome-average. The retention of 3R-ohnologs is even higher for the 2R-ohnologs that belong to family size 3 or 4, and for 2R-ohnologs conserved in all the 27 vertebrates. All the *P*-values are <1e-41, Chi-square test. Family counts are from the intermediate criterion.

Using the expanded OHNOLOGS database we assessed the retention and loss of ohnologs across different vertebrates. We found that on average 25% of extant genes are 2R-ohnologs in vertebrates (intermediate criterion), which include two rounds of WGD, and that 18% of extant genes are 3R-ohnologs in teleost fish, which include an additional WGD (Figure 3A and B; Supplementary Table S4). Teleost fish have also retained more 2R-ohnologs in both absolute numbers (Figure 3A) and relative proportion of extant genes (32% on average). Interestingly, while sauropsids have usually fewer extant genes and 2R-ohnologs than other vertebrates (Figure 3A), they have retained similar or higher proportions of 2R-ohnologs in their genomes (28% on average). Similarly, at the level of individual species, we observe that more compact genomes, such as turkey and tetraodon, which typically contain also fewer genes, have retained about the same numbers and thus larger proportions of ohnologs than other birds or fish, respectively (Supplementary Table S4). This enhanced conservation of ohnologs in individual species or clades with fewer extant genes is consistent with their proposed retention mechanism through purifying selection in paleo-polyploid species (25,27–28).

A vast majority of retained ohnologs consists of protein-coding genes, while non-protein coding genes represent only a small fraction of ohnologs (Supplementary Table S5). For example, in human, out of the 7358 2R-ohnolog pairs from the relaxed criterion only 28 (0.4%) are mi-RNA ohnolog pairs and 2 (0.02%) are sno-RNA ohnolog pairs (Supplementary Table S5).

Remarkably, for all analysed vertebrates the size of 2R-ohnolog families rarely exceeds four ohnologs (Figure 3C), as expected for two rounds of WGD events. Similarly, virtually all 3R-ohnolog families are of size 2, as they are derived from just a single WGD event (Figure 3D). These family sizes also suggest a low rate of small-scale duplications and genome rearrangements following both 2R and 3R-WGD as previously noticed (24).

We then assessed whether teleost fish with their additional 3R-WGD event had further expanded the same ohnolog families as from the previous 2R-WGD events. Indeed, we found that in all four analysed teleost species, 2R-ohnologs tend to retain significantly more 3R-ohnologs (Figure 3E), in agreement with earlier reports (35). The retention of 3R-ohnologs is even higher for 2R-ohnologs that have retained three or four family members, and for the 2R-ohnologs that have been retained in all the 27 vertebrates (Figure 3E). For example zebrafish 2R-ohnologs from the intermediate criteria that have been also retained in all the analysed vertebrates are twice as likely to retain their 3R-ohnologs compared to genome-wide expectation (*P* = 5e-88, Chi-square test). This suggests that the evolutionary mechanism for the expansion of specific gene families through the retention of 2R-ohnologs (25,27–28) might also explain the biased retention of 3R-ohnologs.

**Figure 4.**
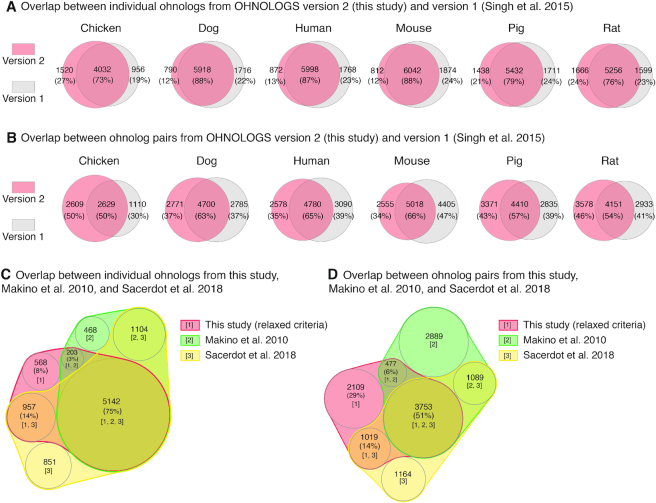
Comparison of ohnologs across different studies. (**A**) Comparison of individual ohnologs from OHNOLOGS v2 (this study) with v1 (34) for the six vertebrates already included in v1. (**B**) Comparison of ohnolog pairs from OHNOLOGS v2 (this study) and v1 (34). The majority of individual ohnologs and pairs are shared between both versions. (**C**) Overlap among individual ohnologs from this study, Makino *etal*. (24) and Sacerdot *etal*. (41). (**D**) Overlap among ohnolog pairs from this study, Makino *etal*. (24) and Sacerdot *etal*. (41). The majority of individual ohnologs and pairs are shared across the three studies. The venn diagram between three sets have been generated using nVenn (42).

We next compared the new OHNOLOGS v2 database (this study) with the 2015 version (v1) (34) to quantify the changes due to the improved pipeline. We noticed that the majority of ohnologs are shared between the two versions for all the six species included in v1 (Figure 4A and B). For example, using the relaxed criterion, 87% of individual ohnologs (Figure 4A) and 65% of ohnolog pairs (Figure 4B) in human were already present in the 2015 version. These differences are due to the improved weighted q-score taking into account the phylogenetically biased sampling of species, a broader taxonomic range and changes in ortholog/paralog relations in the recent Ensembl versions. Indeed, out of 3090 ohnolog pairs not identified in the updated v2 version for human using relaxed criterion (Figure 4B), 36% are filtered out due to poor q-score, 38% due to duplication timing not being at the base of vertebrates, 25% due to changes in orthologs with outgroup genome(s) and 1% due to other Ensembl version related changes. We also compared ohnologs from the current study with ohnologs from Makino *et al.* (24) and Sacerdot *et al.* (41) studies that used different methodological approaches. All these datasets share a significant overlap between them. For example, using the relaxed criterion, 75% of v2 ohnologs are common to the three ohnolog datasets (Figure 4C). We noticed that out of 1089 ohnolog pairs identified by both Makino *et al.* and Sacerdot *et al.* studies but excluded by our analysis (Figure 4D), 56% are filtered out due to poor q-scores and 44% due to changes in ortholog/paralog relationships or Ensembl version related differences. These comparisons suggest that in addition to synteny, identification of the correct timing of duplication and homology relationships are also critical for accurate identification of ohnologs.

At last, the OHNOLOGS v2 database can be used to analyze the branch-specific loss and retention of ohnologs. For instance, we found that 1316 out of 2373 ohnolog families with relaxed confidence criterion in human had an identical size in nearly all the 18 mammals (i.e. corresponding to a variance over mean size ratio lower than 0.1 across all 18 mammals, where ohnolog family sizes are not affected by additional small-scale duplicates). Then, out of these 1316 conserved 2R-ohnolog families in mammals, 702 have an identical size in teleost fish, including 396 families which also share the same size in sauropsids while the remaining 306 families correspond mainly to additional 2R-ohnolog losses in sauropsids; 119 families are larger in teleost fish and contain fish-specific 2R-ohnologs, while 86 families are smaller in teleost fish and correspond to 29 amniota-specific, 49 mammalia-specific and only 8 sauropsida-specific retentions of 2R-ohnologs.

## CONCLUSION

The updated OHNOLOGS v2 database is a comprehensive resource for the genes retained from WGDs across 27 vertebrates. It includes ohnologs from both ancestral vertebrate 2R-WGDs and teleost fish specific 3R-WGD. It is based on a robust pipeline that downloads and processes datasets automatically using Ensembl, which makes it amenable to easy updates. We plan to expand and update OHNOLOGS periodically. Algorithmically, it is based on a quantitative comparative macro-synteny approach, which also takes into account the phylogenetically biased sampling of available vertebrate species. This approach assesses the confidence in each ohnolog pair and robustly identifies ohnologs, despite lineage specific genome rearrangement, gene loss and small-scale duplication events. Using the datasets in OHNOLOGS we show a greater lineage-specific ohnolog loss in sauropids compared to other vertebrate groups, and a high retention of 2R-ohnologs in subsequent 3R-WGD in teleost fish. In the light of the evolutionary significance of ancient WGDs and ohnologs for vertebrate evolution, the expanded and improved OHNOLOGS database should facilitate deeper comparative, evolutionary, genomic and functional analyses of the ohnolog genes in vertebrates.

## DATA AVAILABILITY

All the data and code used to construct OHNOLOGS is available at http://ohnologs.curie.fr and its associated GitHub repository at https://github.com/param-p-singh/Ohnologs-v2.0.

## Supplementary Material

gkz909_Supplemental_Files

## Notes

Present address: Param Priya Singh, Stanford University, Department of Genetics, 300 Pasture Drive, Stanford, CA 94305, USA.

**Disclaimer:** The funders had no role in study design, data collection and analysis, decision to publish, or preparation of the manuscript.
