# Supplementary material for: OHNOLOGS v2: a comprehensive resource for the genes retained from whole genome duplication in vertebrates
Source: Nucleic Acids Res. 2019 Oct 15;48(D1):D724–30. doi: 10.1093/nar/gkz909 (PMC7145513; doi:10.1093/nar/gkz909)
Supplement: gkz909_Supplemental_Files [file gkz909_supplemental_files.zip › Singh-Isambert_OhnologsNAR_SI.pdf]

Supplementary Methods

for manuscript

OHNOLOGS v2: A comprehensive resource for the genes retained from whole genome duplication in vertebrates

Param Priya Singh[1,2] & Hervé Isambert[1]

[1]Institut Curie, Research Center, CNRS UMR168, PSL Research University, 26 rue dUlm, 75005 Paris, France
[2]Present Address: Stanford University, Department of Genetics, 300 Pasture Drive, Stanford, CA 94305, USA

Contents

1 Confidence assessment of ohnolog pairs: combining q-scores 2
1.1 OHNOLOGS v1 database: a simple average of log q-scores over vertebrate species 2
1.2 OHNOLOGS v2 database: a weighted sum of log q-scores over vertebrate species 2

2 Weighting scheme for phylogenetically related sequences 3
2.1 Generic increase of variance due to non-independent samples 3
2.2 Sample weighting scheme by inversion of the variance-covariance matrix 5
2.3 Application to weighting phylogenetically related sequences 6

3 References 7

1 Confidence assessment of ohnolog pairs: combining q-scores

1.1 OHNOLOGS v1 database: a simple average of log q-scores over vertebrate species

The confidence assessment of individual ohnolog pairs in the original OHNOLOGS v1 database (*1*) relied on the definition of quantitative outgroup and self-synteny scores (q-scores) for each vertebrate species, *i.e.* Q^k_{outgroup} and Q^k_{self}, where k =human, mouse, rat, pig, dog and chicken. See Singh *et al.* PLoS Comput Biol 2015 paper (*1*) for a detailed computation of q-scores from synteny comparison.

Then, to circumvent the difficulty to identify ohnolog pairs in each vertebrate genome due to lineage specific rearrangement, gene loss and small scale duplication events, geometric averages of outgroup and self-synteny q-scores were taken over the six amniote species included in the OHNOLOGS v1 database:

$$\log \bar{Q}_{\text{outgroup}} = \sum_{k=1}^{6} \frac{1}{6} \log Q^k_{\text{outgroup}} \quad \text{(S1)}$$

$$\log \bar{Q}_{\text{self}} = \sum_{k=1}^{6} \frac{1}{6} \log Q^k_{\text{self}} \quad \text{(S2)}$$

Using such averaged q-scores was shown to improve the statistical significance of the inferred ohnologs by allowing to identify ohnolog pairs that are no longer in significant synteny in a particular vertebrate genome, if their respective orthologs form a high confidence ohnolog pair in other vertebrates.

However, simple q-score averages fall short of (i) assessing the gain in statistical power expected from the integration of multiple vertebrate species (as the weights 1/6 in Eqs. S1 and S2 sum to 1), as well as, (ii) taking into account the phylogenetically biased sampling of vertebrate species by using equal weights (1/6), while the more recently diverged mouse and rat genomes are expected to bring rather redundant information on ohnolog retention as compared to phylogenetically more distant species such as chicken.

1.2 OHNOLOGS v2 database: a weighted sum of log q-scores over vertebrate species

The expanded OHNOLOGS v2 database addresses the shortcomings on the statistical confidence of ohnolog pairs from the original OHNOLOGS v1 database.

To this end, we modified the definitions of outgroup and self-synteny q-scores, from Eqs. S1 and S2, as weighted sums of log q-scores over all $N = 27$ vertebrate species included in the OHNOLOGS v2 database for 2R-ohnologs and all $N = 4$ included teleost fish species for 3R-ohnologs (see Table S1),

$$\log \bar{Q}_{\text{outgroup}} = \sum_{k=1}^{N} w_k \log Q^k_{\text{outgroup}} \quad \text{(S3)}$$

$$\log \bar{Q}_{\text{self}} = \sum_{k=1}^{N} w_k \log Q^k_{\text{self}} \quad \text{(S4)}$$

where the weights (w_k) are meant to (i) capture the gain in statistical power expected from the integration of 27 vertebrates including 4 teleost fish species (*i.e.* $\sum_k w_k > 1$) and (ii) take into account the strong phylogenetically biased sampling of included species by using different weights for each vertebrate genome depending on its shared homology with other included genomes.

The computation of the individual weights, $w_k^{2\mathrm{R}}$ for 2R-ohnologs and $w_k^{3\mathrm{R}}$ for 3R-ohnologs, are detailed in the following section. It is based on the times of divergence between each pairof vertebrate genomes included in the study (Table S2) and the values of $w_k^{2\mathrm{R}}$ and $w_k^{3\mathrm{R}}$ are listed in Table S3.

The overall gain of statistical power is estimated as $\sum_k w_k^{2\mathrm{R}} \simeq 4.52$ for 2R-ohnologs and $\sum_k w_k^{3\mathrm{R}} \simeq 2.41$ for 3R-ohnologs. This corresponds to an effective number of "independent species" of about 4.5 out of the 27 included vertebrates for assessing the confidence of 2R-ohnologs and to an effective number of "independent species" of about 2.4 out of the 4 included teleost fish for assessing the confidence of 3R-ohnologs.

In addition, as anticipated, recently diverged species of overrepresented vertebrate subgroups are assigned very small weights, which only amount to a very small fraction of the total weight. In particular, each of the 8 included primates has an individual weight around 0.01-0.02, while the sole representatives of long diverged subgroups have proportionally very large weights, such as Spotted Gar ($w \simeq 0.72$) or Anole Lizard ($w \simeq 0.57$).

A consequence of the gain of statistical power between OHNOLOGS v1 and v2 databases is that we could define more stringent confidence criteria for ohnolog pairs and generated ohnolog families as,

- strict $\bar{Q}_{\mathrm{outgroup}} < 0.001$ AND $\bar{Q}_{\mathrm{self}} < 0.001$
- intermediate $\bar{Q}_{\mathrm{outgroup}} < 0.01$ AND $\bar{Q}_{\mathrm{self}} < 0.01$
- relax $\bar{Q}_{\mathrm{outgroup}} < 0.05$ AND $\bar{Q}_{\mathrm{self}} < 0.3$

2 Weighting scheme for phylogenetically related sequences

As discussed above, the effective number N' of "independent species" is smaller than the actual number N of phylogenetically related species included in the analysis.

One way to estimate N' and the corresponding weights w_k for each phylogenetically related species (with $\sum_k^N w_k = N'$) is through the apparent increase of variance of an ordinal character x (such as the number of genome rearrangements) across N non-independent species. The result is quite general and the increase of variance can be used to infer consistent weights for a generic dataset of N non-independent samples, as discussed in the next section.

2.1 Generic increase of variance due to non-independent samples

The generic increase of variance between the N non-independent samples can be illustrated on the example of a theoretical dataset with N' independent samples, each repeated $n_{k'}$ times (or not repeated if $n_{k'} = 1$) to yield a larger dataset of $N = \sum_{k'=1}^{N'} n_{k'}$ non-independent samples.

The variance obtained for the larger non-independent dataset of size N reads:

$$\begin{aligned} V_N &= \frac{1}{N^2}\sum_k^N\sum_\ell^N \langle \delta x^{(k)}\delta x^{(\ell)}\rangle \\ &= \frac{1}{N^2}\sum_{k'}^{N'}\sum_{\ell'}^{N'} n_{k'}n_{\ell'}\langle \delta x^{(k')}\delta x^{(\ell')}\rangle \\ &= \frac{1}{N^2}\sum_{k'}^{N'} n_{k'}^2\langle {\delta x^{(k')}}^2\rangle \\ &= \frac{1}{N^2}\sum_{k}^{N} n_{k}\langle {\delta x^{(k)}}^2\rangle \end{aligned} \tag{S5}$$

as $\langle \delta x^{(k')}\delta x^{(\ell')}\rangle = \delta_{k',l'}\langle {\delta x^{(k')}}^2\rangle$ for independent samples and using $\sum_{k'}^{N'} n_{k'} f(k') \equiv \sum_k^N f(k)$ with $n_k = n_{k'}$ for each of the $n_{k'}$ samples k that are duplicates of sample k'.

When all samples are independent, that is, if $n_k = 1$ for all N samples, one recovers the well known results (adopting the rescaling $\langle {\delta x^{(k)}}^2\rangle = 1$ for all k),

$$V_N = \frac{1}{N^2}\sum_{k}^{N} n_{k}\langle {\delta x^{(k)}}^2\rangle = \frac{1}{N} \tag{S6}$$

By contrast when the samples are not all independent, that is, if $n_k > 1$ for some of the N samples ($\sum_k^N n_k > N$), one gets

$$V_N = \frac{1}{N^2}\sum_{k}^{N} n_{k}\langle {\delta x^{(k)}}^2\rangle = \frac{1}{N_{\mathrm{app}}} > \frac{1}{N} \tag{S7}$$

as if the apparent number of independent samples was smaller, $N_{\mathrm{app}} < N$.

This suggests to weight each non-independent sample k with a probability weight, $w_k = 1/n_k \leqslant 1$ with $\sum_k^N w_k = N'$ and to define the corrected variance for effective sample size as,

$$\begin{aligned} V_{N'} &= \frac{1}{N'^2}\sum_k^N\sum_\ell^N w_k w_\ell\langle \delta x^{(k)}\delta x^{(\ell)}\rangle \\ &= \frac{1}{N'^2}\sum_{k'}^{N'}\sum_{\ell'}^{N'} w_{k'}n_{k'}w_{\ell'}n_{\ell'}\langle \delta x^{(k')}\delta x^{(\ell')}\rangle \\ &= \frac{1}{N'^2}\sum_{k'}^{N'} w_{k'}^2 n_{k'}^2\langle {\delta x^{(k')}}^2\rangle \\ &= \frac{1}{N'^2}\sum_{k'}^{N'} \langle {\delta x^{(k')}}^2\rangle = \frac{1}{N'} \end{aligned} \tag{S8}$$

using $w_k n_k = 1$. Note that $V_{N'}$ can also be expressed in the actual sample space with N non-independent samples, instead of the effective sample space with N' independent samples (which are not typically known), as,

$$
\begin{aligned}
V_{N'} &= \frac{1}{N'^2}\sum_k^N\sum_\ell^N w_k w_\ell \langle \delta x^{(k)} \delta x^{(\ell)} \rangle \\
&= \frac{1}{N'^2}\sum_{k'}^{N'} w_{k'}^2 n_{k'}^2 \langle {\delta x^{(k')}}^2 \rangle \\
&= \frac{1}{N'^2}\sum_{k}^{N} w_{k}^2 n_{k} \langle {\delta x^{(k)}}^2 \rangle \\
&= \frac{1}{N'^2}\sum_{k}^{N} w_{k} \langle {\delta x^{(k)}}^2 \rangle = \frac{1}{N'}
\end{aligned}
\tag{S9}
$$

using $\sum_{k'}^{N'} n_{k'} f(k') \equiv \sum_k^N f(k)$ and $\forall k, w_k n_k = 1$ and $\langle {\delta x^{(k)}}^2 \rangle = 1$.

2.2 Sample weighting scheme by inversion of the variance-covariance matrix

The above results show that the sample weights $\{w_k\}$ are solutions of the following equation,

$$
\sum_k^N\sum_\ell^N w_k w_\ell \langle \delta x^{(k)} \delta x^{(\ell)} \rangle = \sum_k^N w_k \langle {\delta x^{(k)}}^2 \rangle \tag{S10}
$$

While Eq. S10 with N unknown weights is underdetermined, one can easily show that this equation also applies to individual summand for each k as,

$$
\forall k,\ \sum_\ell^N w_\ell \langle \delta x^{(k)} \delta x^{(\ell)} \rangle = \sum_{\ell'}^{N'} w_{\ell'} n_{\ell'} \langle \delta x^{(k)} \delta x^{(\ell')} \rangle = w_k n_k \langle {\delta x^{(k)}}^2 \rangle = \langle {\delta x^{(k)}}^2 \rangle, \tag{S11}
$$

using $\sum_\ell^N f(\ell) \equiv \sum_{\ell'}^{N'} n_{\ell'} f(\ell')$ and $\forall k, w_k n_k = 1$.

Eq. S11 can be written in the following matrix form, after rescaling $\delta x^{(k)}$ by its mean deviation as $\delta x^{(k)}/\sqrt{\langle {\delta x^{(k)}}^2 \rangle}$,

$$
\Sigma \begin{pmatrix} w_1 \\ \vdots \\ w_N \end{pmatrix} = \begin{pmatrix} 1 \\ \vdots \\ 1 \end{pmatrix} \tag{S12}
$$

where $\Sigma = [\Sigma_{k\ell}]$ with $\Sigma_{k\ell} = \langle \delta x^{(k)} \delta x^{(\ell)} \rangle / \sqrt{\langle {\delta x^{(k)}}^2 \rangle}\sqrt{\langle {\delta x^{(\ell)}}^2 \rangle}$ is the rescaled variance-covariance matrix between samples, which leads to the following weight solution whenever the variance-covariance

matrix is invertible,

$$\begin{pmatrix} w_1 \\ \vdots \\ w_N \end{pmatrix} = \Sigma^{-1} \begin{pmatrix} 1 \\ \vdots \\ 1 \end{pmatrix} \tag{S13}$$

While Eq. S13 seems to give the straightforward solution of the generic sample weighting problem, in practice, the variance-covariance matrix Σ is typically not invertible. In particular, straightforward averages of variances and covariances over the available samples, which imply $\sum_k^N \delta x^{(k)} = 0$, yields a singular variance-covariance matrix (as all rows and columns sum to zero).

Yet, in some particular cases, the form of the variance-covariance matrix can be conjectured independently from the specific data of interest and used to solve Eq. S13.

This is the case for time series of dynamical systems with exponential relaxation over time (*2, 3*) or for phylogenetically related sequences (*4, 5*), as discussed in the next section.

2.3 Application to weighting phylogenetically related sequences

The form adopted for the variance-covariance matrix of phylogenetically related sequences is directly inspired by the form proposed by Altschul *et al* in ref. (*5*) to estimate weights of sequence data related by a tree.

Following these authors, we reason that as genome rearrangements and gene loss events accumulated in ancestral vertebrate genomes after each WGD event, the distance of their alignment with the reference paleoploid genome progressively shift. At first approximation, one expect a *linear* accumulation of some finite number (X) of genome rearrangements and gene loss events over time, as these evolutionary changes are essentially non-reversible (small scale duplication events might even lead to exponential growth of gene families over time (*6*)). This is to be contrasted with an unbiased reversible random walk in sequence space, which would lead to a purely diffusive dynamics with a sublinear (square root) accumulations of changes over time.

Hence, due to this progressive shift in genome space, the variance of accumulated changes, X_k, of a given vertebrate genome, G_k, is expected to increase *quadratically* with time t_k since a WGD event, *i.e.* $\langle \delta x^{(k)2} \rangle \sim \sigma^2 t_k^2$, instead of linearly with time for a perfectly diffusive dynamics.

Similarly, the covariance of accumulated changes in two genomes, G_k and G_ℓ, having diverged after some time $t_{k\ell}$ after a WGD event is expected to increase *quadratically* as, $\langle \delta x^{(k)} \delta x^{(\ell)} \rangle \sim \sigma^2 t_{k\ell}^2$, assuming that subsequent changes after the two genomes have diverged were completely independent and could not therefore further increase the covariance.

All in all, this leads to the following form for the rescaled variance-covariance matrix, $\Sigma = [\Sigma_{k\ell}]$, where $\Sigma_{k\ell} = \langle \delta x^{(k)} \delta x^{(\ell)} \rangle / \sqrt{\langle \delta x^{(k)2} \rangle} \sqrt{\langle \delta x^{(\ell)2} \rangle} = t_{k\ell}^2 / t_k t_\ell$, that is independent for the prefactor σ^2.

In the application to compute the weight w_k of each species, the times of 2R-WGD and 3R-WGD were estimated by averaging recent estimates as $t_{2R} = 535$ MY (*7–10*) and $t_{3R} = 328$ MY (*11–15*), respectively, and the times since divergence of each pairs of species, $d_{k\ell} = t_{WGD} - t_{k\ell}$, were taken from the TimeTree database (*16*) and listed in Table S2. The final values for 2R- and 3R-WGD weights are listed in Table S3.

3 References

1. Singh PP, Arora J, Isambert H (2015) Identification of Ohnolog Genes Originating from Whole Genome Duplication in Early Vertebrates, Based on Synteny Comparison across Multiple Genomes. *PLoS Comput. Biol.* 11(7):e1004394.

2. Jones RH (1975) Estimating the variance of time averages. *J. Appl. Meteor.* 14(2):159–163.

3. Verny L, Sella N, Affeldt S, Singh PP, Isambert H (2017) Learning causal networks with latent variables from multivariate information in genomic data. *PLoS Comput. Biol.* 13(10):e1005662.

4. Felsenstein J (1973) Maximum-likelihood estimation of evolutionary trees from continuous characters. *Am. J. Hum. Genet.* 25(5):471–492.

5. Altschul SF, Carroll RJ, Lipman DJ (1989) Weights for data related by a tree. *J. Mol. Biol.* 207(4):647–653.

6. Evlampiev K, Isambert H (2008) Conservation and topology of protein interaction networks under duplication-divergence evolution. *Proc. Natl. Acad. Sci. U.S.A.* 105(29):9863–9868.

7. Putnam NH, et al. (2008) The amphioxus genome and the evolution of the chordate karyotype. *Nature* 453(7198):1064–1071.

8. Holland LZ, et al. (2008) The amphioxus genome illuminates vertebrate origins and cephalochordate biology. *Genome Res.* 18(7):1100–1111.

9. Van de Peer Y, Maere S, Meyer A (2009) The evolutionary significance of ancient genome duplications. *Nat. Rev. Genet.* 10(10):725–732.

10. Smith JJ, Keinath MC (2015) The sea lamprey meiotic map improves resolution of ancient vertebrate genome duplications. *Genome Res.* 25(8):1081–1090.

11. Jaillon O, et al. (2004) Genome duplication in the teleost fish Tetraodon nigroviridis reveals the early vertebrate proto-karyotype. *Nature* 431(7011):946–957.

12. Hoegg S, Brinkmann H, Taylor JS, Meyer A (2004) Phylogenetic timing of the fish-specific genome duplication correlates with the diversification of teleost fish. *J. Mol. Evol.* 59(2):190–203.

13. Christoffels A, et al. (2004) Fugu genome analysis provides evidence for a whole-genome duplication early during the evolution of ray-finned fishes. *Mol. Biol. Evol.* 21(6):1146–1151.

14. Vandepoele K, De Vos W, Taylor JS, Meyer A, Van de Peer Y (2004) Major events in the genome evolution of vertebrates: paranome age and size differ considerably between ray-finned fishes and land vertebrates. *Proc. Natl. Acad. Sci. U.S.A.* 101(6):1638–1643.

15. Hurley IA, et al. (2007) A new time-scale for ray-finned fish evolution. *Proc. Biol. Sci.* 274(1609):489–498.

16. Hedges SB, Marin J, Suleski M, Paymer M, Kumar S (2015) Tree of life reveals clock-like speciation and diversification. *Mol. Biol. Evol.* 32(4):835–845.
